# Electrostatic shock acceleration of ions in near-critical-density plasma driven by a femtosecond petawatt laser

**DOI:** 10.1038/s41598-020-75455-1

**Published:** 2020-10-28

**Authors:** Prashant Kumar Singh, Vishwa Bandhu Pathak, Jung Hun Shin, Il Woo Choi, Kazuhisa Nakajima, Seong Ku Lee, Jae Hee Sung, Hwang Woon Lee, Yong Joo Rhee, Constantin Aniculaesei, Chul Min Kim, Ki Hong Pae, Myung Hoon Cho, Calin Hojbota, Seong Geun Lee, Florian Mollica, Victor Malka, Chang-Mo Ryu, Hyung Taek Kim, Chang Hee Nam

**Affiliations:** 1 Center for Relativistic Laser Science, Institute for Basic Science (IBS), Gwangju, 61005 Republic of Korea; 2 Advanced Photonics Research Institute, Gwangju Institute of Science & Technology (GIST), Gwangju, 61005 Republic of Korea; 3 Department of Physics and Photon Science, GIST, Gwangju, 61005 Republic of Korea; 4 Amplitude Laser Group, 91090 Lisses, France; 5 Laboratoire D’Optique Appliquée, ENSTA-ParisTech, Ecole Polytechnique, 828 Boulevard des Marechaux, 91762 Palaiseau CEDEX, France; 6 Weizmann Institute of Science, P.O. Box 26, 76100 Rehovot, Israel

**Keywords:** Astrophysical plasmas, Laser-produced plasmas

## Abstract

With the recent advances in ultrahigh intensity lasers, exotic astrophysical phenomena can be investigated in laboratory environments. Collisionless shock in a plasma, prevalent in astrophysical events, is produced when a strong electric or electromagnetic force induces a shock structure in a time scale shorter than the collision time of charged particles. A near-critical-density (NCD) plasma, generated with an intense femtosecond laser, can be utilized to excite a collisionless shock due to its efficient and rapid energy absorption. We present electrostatic shock acceleration (ESA) in experiments performed with a high-density helium gas jet, containing a small fraction of hydrogen, irradiated with a 30 fs, petawatt laser. The onset of ESA exhibited a strong dependence on plasma density, consistent with the result of particle-in-cell simulations on relativistic plasma dynamics. The mass-dependent ESA in the NCD plasma, confirmed by the preferential reflection of only protons with two times the shock velocity, opens a new possibility of selective acceleration of ions by electrostatic shock.

## Introduction

Collisionless shocks are observed in various astrophysical phenomena, such as cosmic ray, gamma-ray burst and active galactic nuclei^1–3^. The collisionless shock of an electromagnetic type is associated with strong magnetic filaments, formed by Weibel^4^ or current filamentation instability^4^, whereas the shock of electrostatic nature is typically formed by ion-acoustic wave excitation^5–8^. An electrostatic shock can also be an effective tool in achieving energetic ion beams in laser-driven charged particle accelerators^9–13^. The electrostatic shock acceleration (ESA) in plasmas produced with nanosecond or sub-nanosecond lasers mostly develop longitudinally, i.e., the shock wave propagates along the laser propagation direction^9,14–16^.

In general, to observe the scaled-down astrophysical scenarios in a laboratory for understanding plasma shocks and instabilities occurring on the time-scale where ion dynamics is crucial, nanosecond or picosecond long laser pulses are used to continuously pump the energy into the plasma such that instabilities can be sustained over a significant time period^17^. Most often, the details of astrophysical collisionless shock phenomena can remain inaccessible due to the limitations in astronomical observations. A scaled-down version of shock formed by laser-driven plasmas can, however, allow us to explore the underlying physics of the astrophysical shocks, like the observation of turbulent collisionless shocks in conditions relevant to young supernova remnants in the laser-driven plasma flow experiments^18^. Recent numerical and experimental studies with underdense plasma^19,20^ and short but intense laser pulses^21–27^, however, showed the excitation of strong electrostatic sheath field on the channel wall and consequent transverse ion acceleration, which leads to an expectation that with an ultrahigh intensity femtosecond laser the electrostatic shock can be excited in plasma to reflect upstream ions via shock-acceleration.

Here, we applied, for the first time, a femtosecond petawatt (PW) laser to the generation of near-critical-density (NCD) plasmas for forming a collisionless electrostatic shock structure that accelerates ions transversally. Thanks to the technological advancement of ultrashort and high power lasers, ultrahigh power lasers with outputs exceeding one PW have been constructed or being developed in several laser facilities around the world^28^. At the Center for Relativistic Laser Science, Institute for Basic Science, two PW laser beamlines have been constructed and utilized for the investigations of laser-driven charged particle acceleration and ultrahigh intensity laser-plasma interactions^29^. The choice of an NCD plasma helps achieve larger coupling efficiency of the laser to a bulk plasma than what can be achieved in an underdense or overdense plasma. The production of a relativistic plasma by applying a PW laser pulse with relativistic intensity brings interesting consequence in plasma dynamics. The expulsion of relativistic electrons turns out to be crucial in sustaining the electrostatic field in the plasma long enough for the formation of a transverse shock, even with a 30 fs laser driver, because in the relativistic plasma the plasma response time is slowed down due to the relativistic mass increase of electrons.

A most interesting aspect related to ESA is the dependence on ion mass. In ESA, upstream ions are reflected back when the potential energy across a shock wall exceeds the kinetic energy of upstream ions. In the case of a multi-species plasma only those stationary upstream ions, satisfying the condition, ${m}_{i} <2\frac{{{q}_{i}\varphi }_{s}}{{v}_{s}^{2}}$, with mass ${m}_{i}$, charge ${q}_{i}$, potential ${\varphi }_{s}$, and the shock velocity *v*_*s*_, can be reflected back by the shock. To show the mass dependence of the ESA, an NCD plasma was formed by ionizing a high-density helium gas jet, containing a small fraction of hydrogen. It was shown that a transverse electrostatic shock in the NCD plasma preferentially reflected only light protons, rather than bulk heavier helium ions, demonstrating mass-dependent shock acceleration. To examine the shock formation and acceleration processes, we carried out multi-dimensional particle-in-cell (PIC) simulations with the OSIRIS^30,31^ and compared with the experiments performed with a femtosecond PW laser^29^. Our observations demonstrated that such strong laser-bulk plasma interaction in a subcritical density medium can excite electrostatic shock, which opens another branch of applications for ultrashort, ultra-intense lasers to study collisionless shocks in laboratory-scale environments^32^.

## Results

### Experimental realization of NCD plasma by a PW laser

**Figure 1:**
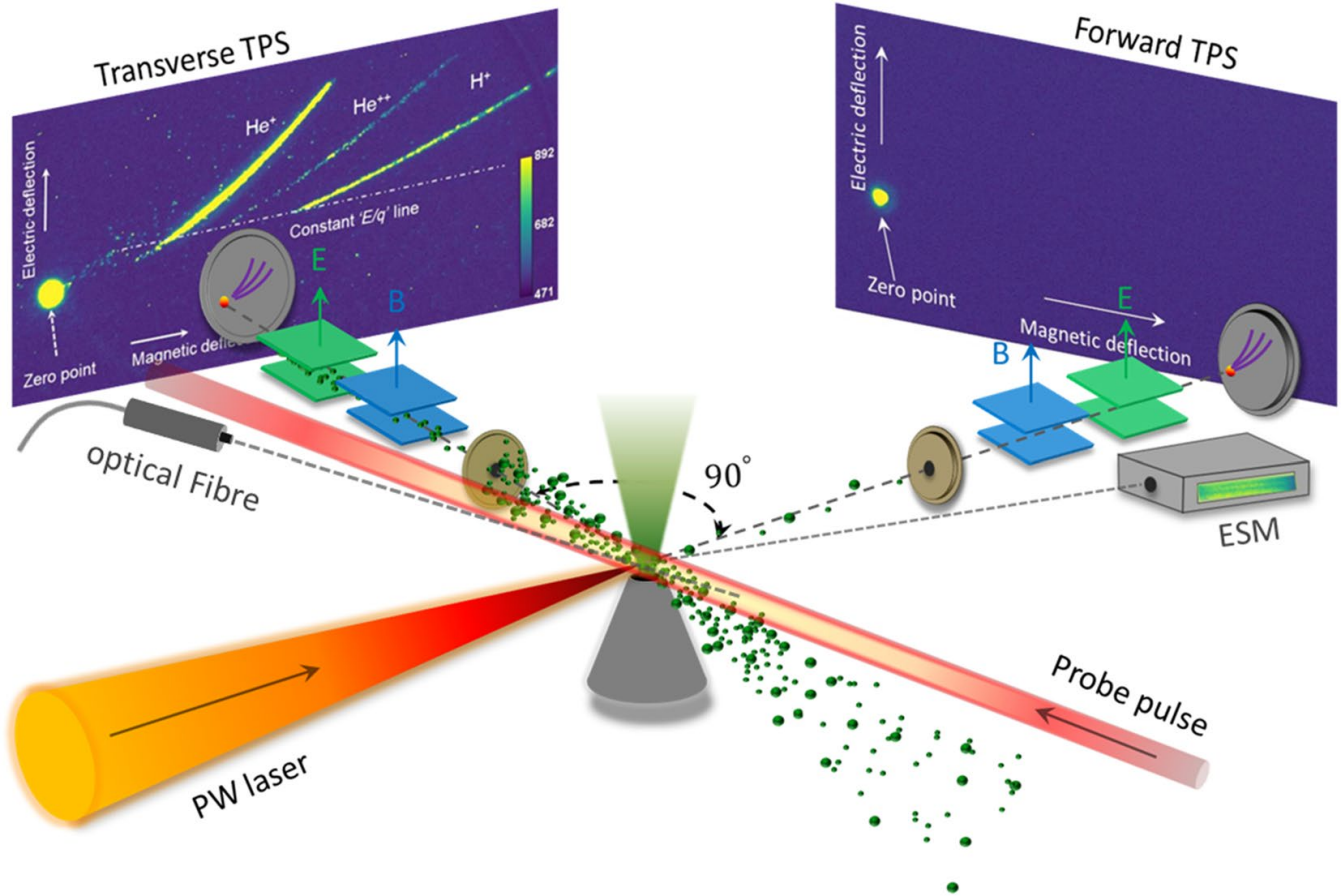
Experimental setup: NCD plasma produced by focusing 1-PW, 30-fs pump laser onto a high-density supersonic gas jet. A 35 fs, 800 nm probe pulse records the plasma shadowgram at different time delays. Two Thomson parabola spectrometers (TPS) were installed for ion energy spectra in the forward and the transverse directions and an electron spectrometer (ESM) for electron energy spectra. An optical fiber spectrometer recorded the Raman scattering signal from the plasma.

The transverse shock acceleration in an NCD plasma was investigated experimentally in a bulk helium plasma, mixed with a small fraction of hydrogen as test particles, excited by a femtosecond PW laser^29^. For the realization of the shock acceleration, a PW laser pulse with 30 fs duration at 800 nm was focused with a spherical mirror onto a He gas jet with an intensity of $1\times {10}^{20}$ W cm^−1^ (${a}_{0}=7$), as shown in the experimental setup in Fig. 1. The He gas was produced by a high-density gas jet (Source Lab SL-GT-10), having a nozzle with 400-µm diameter^33^. By changing the nozzle height with respect to the laser focus, the electron plasma density was scanned in the range of ${n}_{e}=2\times {10}^{20}-4\times {10}^{20}$ cm^−3^ (Supplementary Fig. S1a). During the laser plasma interaction, the Raman scattering signal was also measured (Supplementary Fig. S1b). The plasma density estimated from the side Raman scattering (${n}_{e}=2\times {10}^{20}$ cm^−3^) showed a good agreement with the density estimated by the neutral gas density measurement. A 35-fs probe pulse at 800 nm, propagating orthogonally to the PW laser beam, was used to measure the plasma shadowgram at different time delays (Fig. 1).

**Figure 2:**
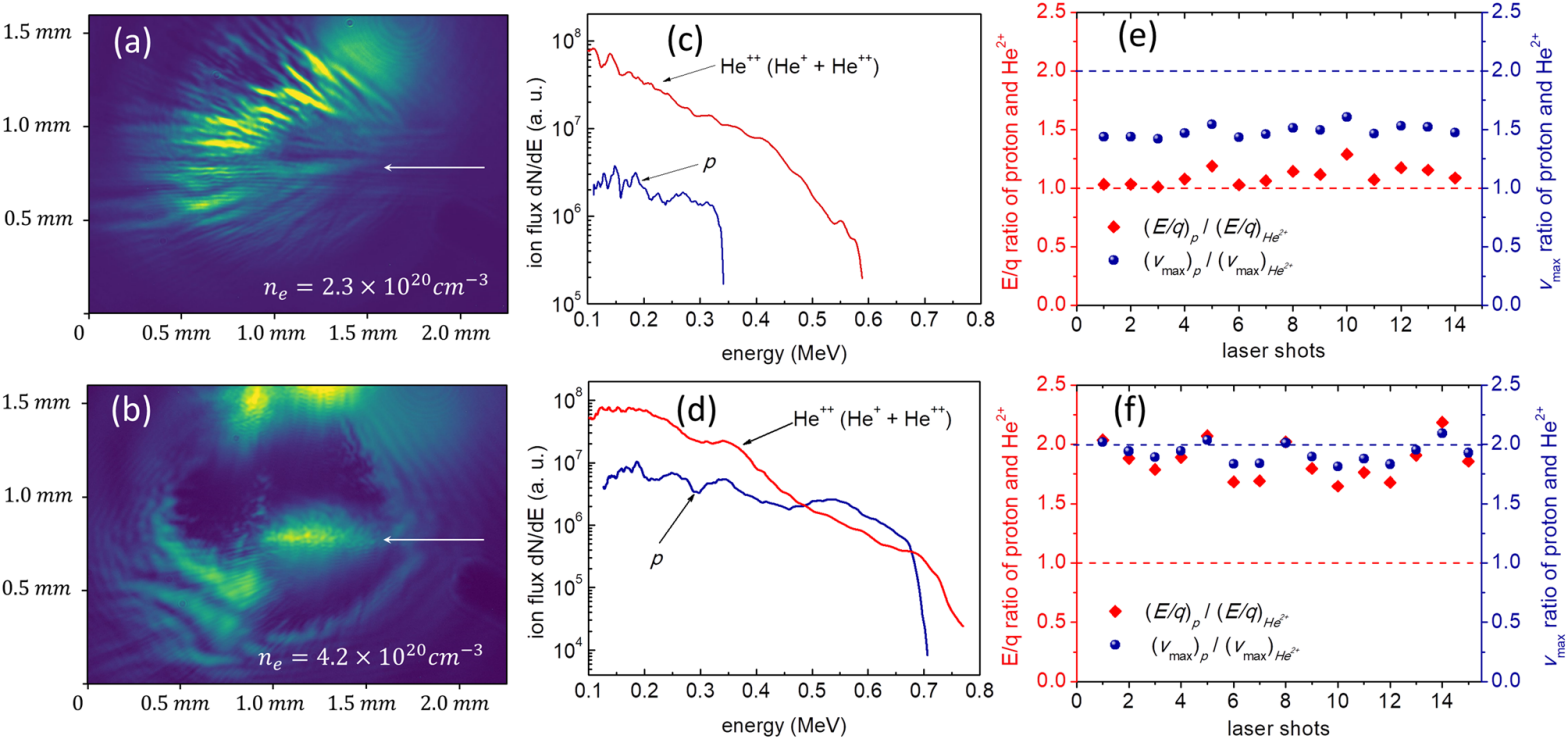
Plasma density-dependent features of ion acceleration: (**a**, **b**) shadowgram images of plasma at a temporal delay of 20 ps. The white arrow indicates the laser propagation direction. The bright light ahead of the arrow originates from the plasma scattered light. (**c**, **d**) Kinetic energy spectra of helium and protons. In (**e**, **f**) the ratio of kinetic energy (*E*) per charge (*q*) between proton and helium in red and ratio of maximum velocity (*v*_*max*_) between proton and helium in blue are shown at different plasma densities. (**a**, **c**, **e**) correspond to the plasma density of *n*_*e*_ = 2.3 × 10^20^ cm^−3^, whereas (**b**, **d**, **f**) correspond to higher plasma density *n*_*e*_ = 4.2 × 10^20^ cm^*−*3^. The two bright spots on the top section of (**b**) originate from the reflection from the nozzle tip.

The kinetic energy spectra of ions were measured with Thomson parabola spectrometers (TPS) along the forward (0°) and the transverse (90°) directions to the laser propagation. Parabolic ion traces, dispersed by the magnetic field (0.2 T) and the electric field (2 kV/cm), were recorded using a micro-channel plate (MCP) with a phosphor screen imaged by a 16-bit CCD camera. The MCP-Phosphor screen-CCD system was calibrated for protons with energy up to 6.2 MeV by installing slotted CR-39 track detectors in front of the MCP. During the experiment, no noticeable forward ion traces were recorded above the detection threshold of 180 keV for He ions (Fig. 1). On the other hand, along the transverse direction both charge states of helium ion (He^+^ and He^2+^) were detected in most shots. Moreover, the proton trace, present in the helium plasma, with a maximum energy of 0.5  MeVwas also observed. The traces for all three ions can be seen in the transverse TPS, as shown in Fig. 1. The helium ion (He^2+^) spectrum in Fig. 2 was obtained by adding the spectra of both He^+^ and He^2+^ since He^+^ originated from the charge exchange process^34^ of He^2+^ (details are given in Supplementary Sect. B). The inclusion of neutral helium in the combined spectrum was not necessary, because, in the high-energy regime, the fraction of neutral helium from the charge exchange process is very small (Fig. S2b).

In addition, relativistic electrons with energy up to 20 MeV were also detected along the forward direction at an angle 10° (measurement are shown in Supplementary Fig. S1c). The observed electron temperature of 3  MeV from the experiment and 3.75  MeV from the 2D PIC simulation (see Supplementary Fig. S1c,d), suggest that the interaction of intense laser pulse with NCD plasma increases the electron inertia by the relativistic factor, $\gamma =6,$ which in effect slows down the plasma response time (${\omega }_{pe}^{-1})$. Therefore, the acceleration of electrons to relativistic energy facilitates the formation of the long-lasting electrostatic field in the plasma channel necessary for the formation of a transverse shock even with a 30 fs laser pulse*.*

### Features of plasma-density-dependent ion acceleration in the experiment

One of the crucial experimental results was the dependence of laser-plasma interaction on plasma density. Figure 2a,b show the shadowgrams of the interaction, captured at the delay of 20 ps, corresponding to the plasma densities of $2.3\times {10}^{20} {\mathrm{cm}}^{-3} (0.13 {n}_{c})$ and $4.2\times {10}^{20}{\mathrm{ cm}}^{-3}$ $(0.24 {n}_{c})$, respectively. The detailed plasma dynamics is given in the Supplementary (Fig. S3). For the lower plasma density, the laser absorption is quite extended and multiple plasma filaments are observed (Fig. 2a). On the other hand, for the higher plasma density, the laser energy depletion occurred in a very small volume, indicated by a much smaller and brighter plasma scattering image (Fig. 2b). The observation of concentrated strong scattering of the driving laser, visible even without a probe beam (see the laser scattering part in Fig. 2b), is consistent with the localized energy deposition of the laser pulse in the high-density plasma. The localized absorption of laser energy created a high-energy–density region, along with rapid evacuation of electrons, and later on, followed by ions. Due to this localized energy deposition, nearly circular cloud expansion is seen in the image.

The plasma-density dependent behaviour of channel formation also affects the subsequent ion acceleration process. In the low-density case, the maximum energy of the proton is two times smaller than helium ion (Fig. 2c), whereas, in the high-density case, the maximum energies are nearly identical (Fig. 2d). A summary of these features is shown in Fig. 2e,f, where the ratios of two parameters, namely “energy-per-charge” and “maximum velocity” for proton and helium ion (${He}^{2+}$) are plotted. In the low-density case, the ratio of energy-per-charge (red diamond) is consistently close to unity for consecutive laser shots. This result, thus, shows that during the acceleration process most energetic hydrogen and helium ions were produced by the similar electrostatic potential formed by the charge separation between ions and electrons since the net energy gain by ions in the same electrostatic potential is proportional to ion charge.

In the high-density regime (4.2 × 10^20^ cm^−3^), the acceleration dynamics of ions is very different from that of the low-density case. The ratio of the maximum-energy-per-charge of the proton to He^2+^ is close to two for all the shots shown in Fig. 2f. This indicates that the velocity of the proton is twice that of He^2+^, which agrees with the shock reflection of protons from the expanding channel of helium ions (Fig. 2b). The shadowgram clearly shows the formation of a near-spherical or oval plasma structure, expanding with the speed of $0.02c$. The kinetic energy of helium ions flying with the expanding plasma channel at the speed of $0.02c$ is 0.75  MeV, comparable to the maximum energy observed in the spectrum (Fig. 2d). On the other hand, the maximum proton energy of 0.7  MeV in Fig. 2d corresponds to the proton speed of $0.04c$. Consequently, this observation indicates that in the presence of an expanding plasma wall structure, the protons are reflected from this wall with twice the expanding speed, while the helium ions move with the expanding wall.

### Transverse dynamics of channel wall and formation of collisionless shocks in PIC simulation

To understand the basic mechanism of transverse shock formation and consequent shock acceleration, two-dimensional (2D) PIC simulations are carried out with three species, (1) electron, (2) He^2+^ and (3) proton. A fixed simulation box (x–z) with perfectly absorbing boundary conditions, and dimensions $1000c/{\omega }_{0}$×$2000c/{\omega }_{0}$ is divided into 5000 × 10,000 cells, where ${\omega }_{0}$ is the angular frequency of the laser field, which is propagating in the z-direction. A cold plasma of three species; electron (charge density: 0.0505n_cr_), He^2+^ (charge density: 0.05n_cr_), and proton (charge density: 0.0005n_cr_), was initialized with 4 × 4 particles-per-cell in each direction for each species. The plasma was initialized at $z=130c/{\omega }_{0}$ with an initial linear density ramp reaching 0.0505n_cr_ at $z=200c/{\omega }_{0}$ and remains constant onwards. A linearly polarized (along y-direction) Gaussian laser beam with FWHM pulse length $60c/{\omega }_{0}$ (${\tau }_{L}=30 \mathrm{fs}$), transverse spot size r_L_ = $80c/{\omega }_{0}$ (12 μm FWHM) and peak normalized vector potential ${a}_{0}=7$ was initialized at z = $130c/{\omega }_{0}$.

**Figure 3:**
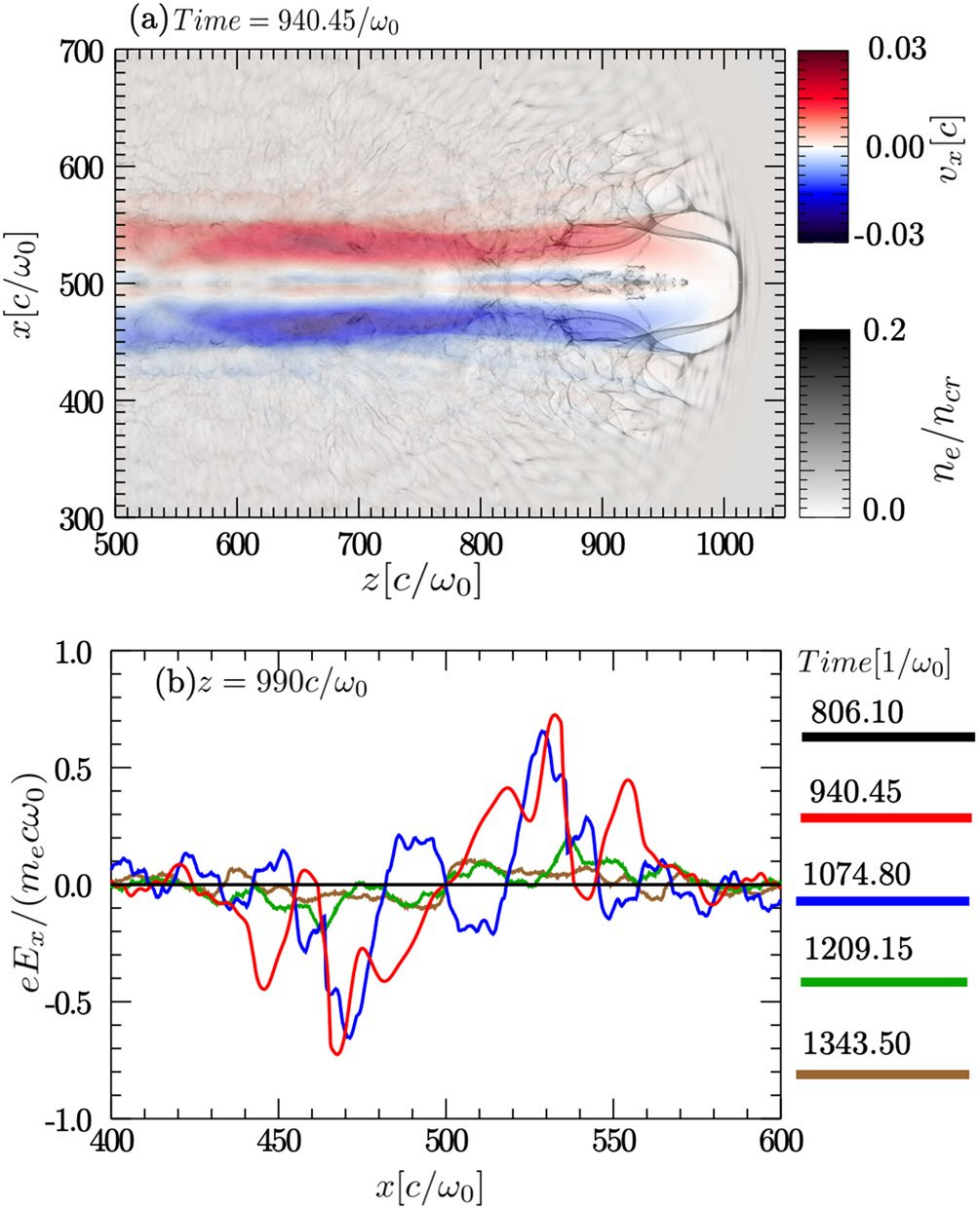
Ion motion within the space charge field created by the laser pulse. (**a**) Snapshot of the electron density profile (grey color scale), superimposed with the transverse velocity distribution of ${He}^{2+}$ (red–white–blue color scale) at time $940.45{/\omega }_{0}$. (**b**) Transverse field distribution along the x directions at z = $990c{/\omega }_{0}$ at various times. The propagation direction of the laser pulse is from left to right. The central axis of the laser pulse is located at $x = 500c/{\omega }_{0}$.

As the high-intensity laser pulse propagates through the helium plasma, the evacuation of electrons by the relativistic intense laser pulse creates a strong-space charge field due to immobile ions in the time scale ${\approx \omega }_{p}^{-1}$, where ${\omega }_{p}$ is electron plasma frequency^35^. Due to the inertia of relativistic electrons, the transient space charge can survive on the time scale ${\sim \omega }_{pi}^{-1}$ ≫ ${\omega }_{p}^{-1}$, where ${\omega }_{pi}$ is ion plasma frequency. Consequently, the ions, experiencing space-charge field, gain transverse momentum with inhomogeneous velocity distribution as shown in Fig. 3a, leading to ballistically focused ion density spike at the edge of laser focus and creates a channel wall, exciting a strong bipolar field^36^.

A 2D snapshot of electron density distribution (grey color scale) along with the transverse velocity distribution for the ${He}^{2+}$ ions (red-white-blue color scale) at time $t=940.45 {\omega }_{0}^{-1}$, is shown in Fig. 3a, and respective transverse electrostatic field ${(E}_{x}$) along the transverse direction at fixed position z = $990c{/\omega }_{0}$ is plotted at various times in Fig. 3b to highlight that the space charge created just behind the laser by the expulsion of electrons survives on time scales similar to ion response time, which is roughly 120 fs for 0.05n_cr_ helium plasma. After arrival of the laser pulse, a strong, axis-symmetric ${E}_{x}$ field is excited ($t=940.45 {\omega }_{0}^{-1}$), which drives the transverse acceleration of helium ions from the plasma channel (Fig. 3a). The noticeable field strength can be observed up to time $t=1209.15 {\omega }_{0}^{-1}$ (Fig. 3b), which suggest that the space-charge fields survive for minimum 120 fs. The survival of the ${E}_{x}$ field in the plasma channel to such long time scales is due to the relativistic inertia of electrons, which turns out to be a crucial factor in facilitating the transverse acceleration of ions from the plasma channel, driven by an intense laser pulse of duration as short as 30 femtosecond.

**Figure 4:**
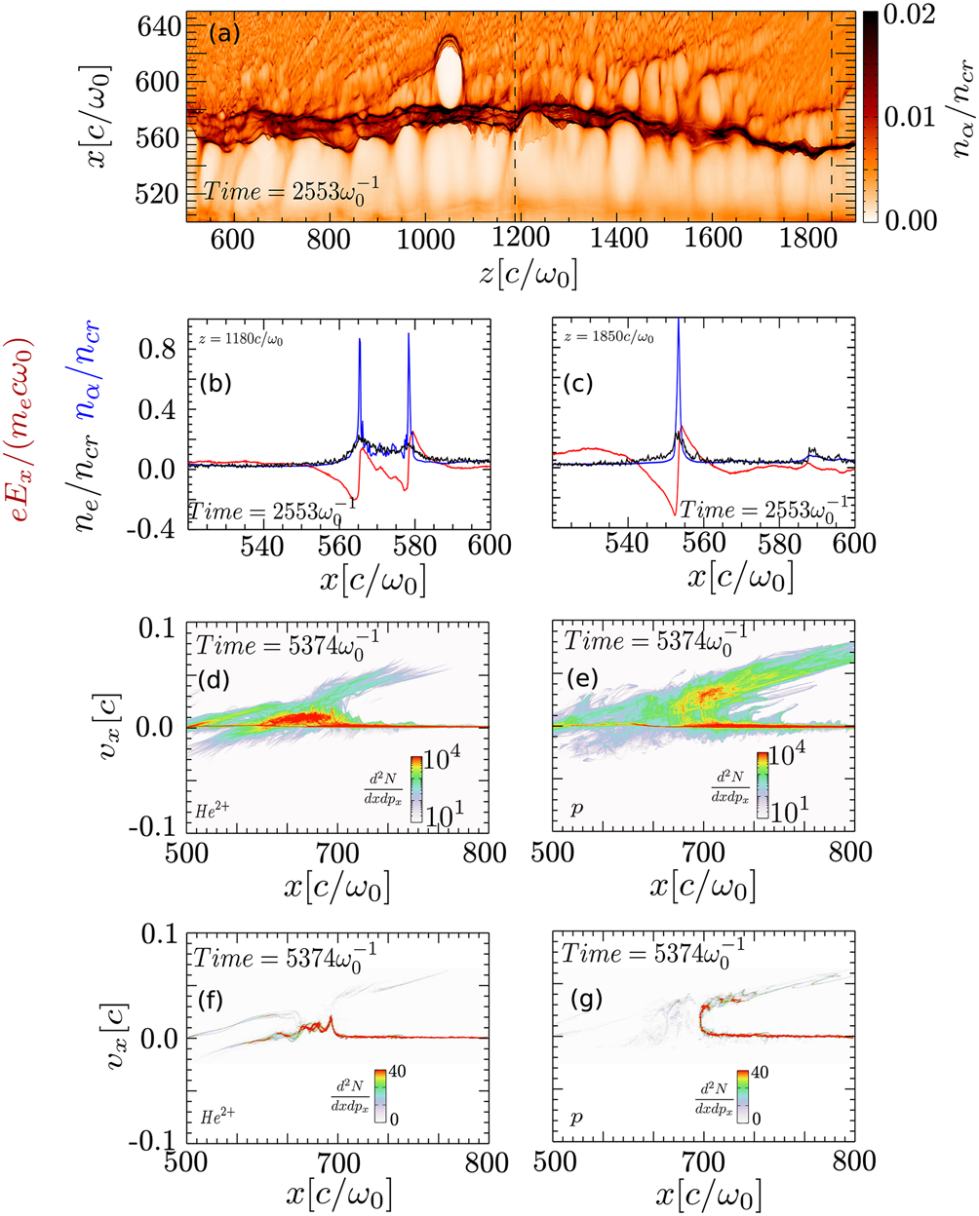
Transverse dynamics of channel wall and formation of collisionless shocks: (**a**) Snapshot of the upper half ${He}^{2+}$ density channel at t = 1.1 ps to show an early stage of shock formation. The direction of laser pulse propagation is from left to right. The central axis of the laser pulse is located at $x = 500c/{\omega }_{0}$. (**b**, **c**) Profiles of electron density (black), helium ion density (blue) and transverse electric field (red) at two locations, (b) $z = 1180 c/{\omega }_{0}$ and (c) $z = 1850 c/{\omega }_{0}$, indicated by the two black dotted lines in (**a**). Transverse phase-space of ${He}^{2+}$ (**d**, **f**) and protons (**e**, **g**) at t = 2.2 ps is plotted, where to plot (**f**, **g**) only particles within longitudinal range $z = 1200 c/{\omega }_{0}$ to $1250 c/{\omega }_{0}$ is used. Here ${\omega }_{0}^{-1}=0.42 fs$ and $c/{\omega }_{0}=0.13 \mathrm{\mu m}$.

In Fig. 4a, as the laser pulse propagates from left to right, the z-axis can be seen as a reversed time axis, i.e., plasma evolution in the rightmost side ($z = 1800 c/{\omega }_{0})$ is at an earlier stage than in the leftmost ($z = 600 c/{\omega }_{0})$. Around the central laser axis ($500c/{\omega }_{0} <x < 550c/{\omega }_{0}$), the low helium ion density, ${n}_{\alpha }\approx 0.02{n}_{cr}$, shows the evacuation of ${He}^{2+}$ and the formation of the channel wall around $x=550c/{\omega }_{0}$. In the early phase of channel formation, a single bipolar field structure is formed, caused by the locally unbalanced screening of ions from thermal background electrons (Fig. 4c). At later stages, the single wall structure splits into a multi-layer wall (Fig. 4b), creating an electrostatic shock structure propagating with shock velocity ${v}_{s}\approx 0.016c$. The origin of such splitting is linked with ion wave breaking dynamics where the faster ions overtake the slower ions in the front^36–38^. The details of the shock splitting will be discussed in the following theoretical paper because many aspects of plasma dynamics should be discussed to understand it comprehensively. We also observe few localized soliton structures on the wall (z = $1050c/{\omega }_{0}$), similar to observed by Sarri et al.^39^.

**Figure 5:**
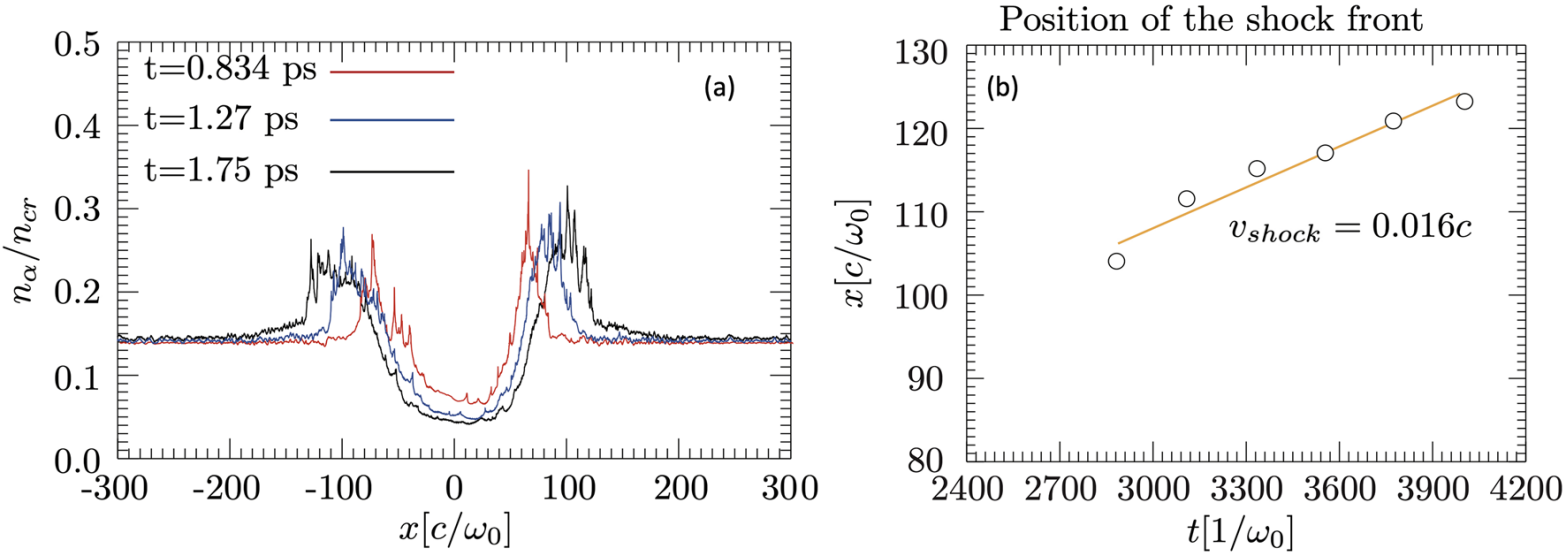
Temporal evolution of shock: (**a**) temporal evolution of the shock wall around the plasma channel. (**b**) Estimation of the shock front velocity.

To estimate shock velocity, the transverse evolution of ion density averaged over a small plasma-size was followed in time. One example is shown in Fig. 5a, where, for the simulation results discussed in Figs. 3 and 4, the average ion density in z over a small plasma block from $z=1100c/{\omega }_{0}$ to $z=1200c/{\omega }_{0}$ is plotted for various times to show that the shock front is moving with velocity $0.016c$ (Fig. 5b).

### Mass-dependent selective electrostatic shock acceleration of the proton

The selective shock acceleration of light protons over massive helium ions can be understood from the phase space plots shown in Fig. 4d–g. Figure 4d,e show the phase-space distribution of proton and helium ions respectively including all the particles in the simulation box, after the evolution of the electrostatic shock. As we have seen from Fig. 4a,c, plasma at different longitudinal position goes through a different dynamic phase. Hence at a given time, to clearly distinguish the sign of shock acceleration, in Fig. 4f,g we plot the transverse phase space of ${He}^{2+}$ and protons for a limited longitudinal range of $z = 1200 c/{\omega }_{0}$ to $1250 c/{\omega }_{0}$. Majority of ${He}^{2+}$ are located around the transverse region of $x = 600 c/{\omega }_{0},$ which corresponds to the position of the channel wall at that time. A significant fraction of He^2+^ ions are part of the transverse shock which is propagating with speed of ${v}_{s} \approx 0.016c$. As most of the background particles ahead of the channel wall have zero momentum (Fig. 4d,e), in the reference frame of the channel wall these ions are moving towards the wall with the shock speed. For background ions to be reflected by the shock front, the potential energy (${{q}_{i}\times \varphi }_{s}$) across the shock wall should be higher than the kinetic energy ($0.5{m}_{i}{v}_{s}^{2}$) of the ions moving with the wall speed. Thus, for ${He}^{2+}$ and proton, the electric potential should be greater than $0.4 {m}_{e}{c}^{2}/e$ and $0.2 {m}_{e}{c}^{2}/e$, respectively. In the simulation, once the collisionless electrostatic shock is formed, the potential at the shock front is calculated to be around ${\varphi }_{s}\approx 0.6 {m}_{e}{c}^{2}/e$ (Fig. 4b), and decays gradually with time. At time t = 5374 ${\omega }_{0}^{-1}$, the potential reduces to ${\varphi }_{s}\approx 0.3 {m}_{e}{c}^{2}/e$, which is too small to reflect helium ions, but still large enough to reflect protons. Consequently, during the electrostatic wall expansion, the potential across the wall decreases and finally becomes too weak to reflect upstream helium ions; however, it remains strong enough to reflect protons. The phase space plot does not show any significant ${He}^{2+}$ ion population moving with twice the speed of the shock wall (Fig. 4d,f). A small fraction of ${He}^{2+}$ particles are accelerated with higher energy, but they are accelerated during the x-wave breaking process when the single-layer breaks into the multi-layer bipolar field structure^36^.

On the other hand, the background protons can be reflected back with twice the velocity of the shock (see Fig. 4e,g), since the potential ${\varphi }_{s}$ remains larger than $0.2 {m}_{e}{c}^{2}/e$ in the calculation. Here in contrast to massive helium ions, a dominant trace of protons, around ${{v}_{H} =2 v}_{s} = 0.03c$, can be seen located just ahead of helium shock structures at $x = 600 c/{\omega }_{0}$, showing the ion-reflection occurring for light particles by the shock wall. The simulation results, consequently, show that massive helium ions contribute to the formation of transverse electrostatic shock, without significant reflection of upstream helium ions, while light protons are easily reflected from the shock wall, confirming the mass-dependent shock acceleration.

### Density-dependent physics of particle acceleration observed in PIC simulation

**Figure 6:**
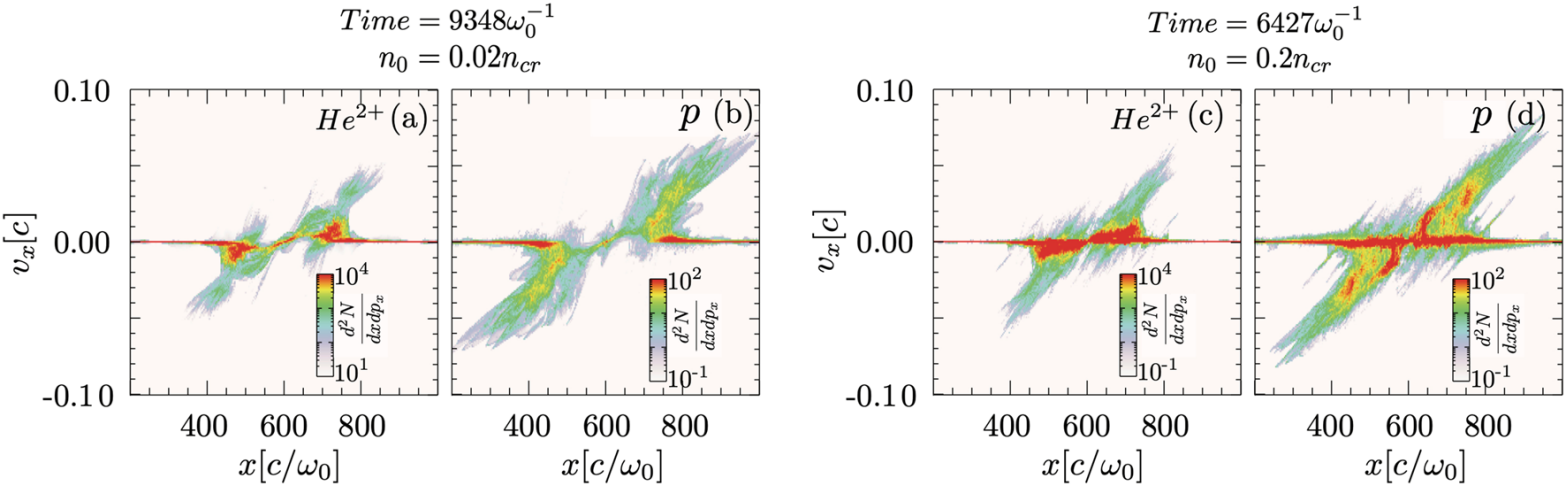
Density-dependent particle acceleration: Phase space plot of helium (**a**, **c**) and proton (**b**, **d**) at time and density of (**a**, **b**) $t=9348 {\omega }_{0}^{-1}$ and ${n}_{0}=0.02{n}_{cr}$, and (**c**, **d**) $t=6427{\omega }_{0}^{-1}$ and ${n}_{0}=0.2{n}_{cr}$. The phase space plots for the two cases are shown for different times since plasma dynamics is density-dependent.

The density-dependent nature of particle acceleration, observed in the experiments, was confirmed in PIC simulations, where additional simulations were performed with density profiles similar to the density profiles in the experiment. A fixed simulation box (x–z) with dimensions $1200c/{\omega }_{0}\hspace{0.17em}$× $6000c/{\omega }_{0}$ is divided into 6000 × 28,000 cells, with laser field of same parameters as in Figs. 3 and 4 propagating in the z-direction. Three species, electron, He^2+^, and proton 1% by density, with same Gaussian density profiles, were initialized with 4 × 4 particles-per-cell in each direction for each species. The Gaussian plasma profile was initialized at $z=150c/{\omega }_{0}$, reaching peak density at $z=4400c/{\omega }_{0}$. The acceleration of the bulk helium ions does not show plasma density dependence. For instance, by changing the plasma density from 0.02 ${n}_{cr}$ (Fig. 6a) to 0.2 ${n}_{cr}$ (Fig. 6c), the phase space plot of helium does not have any shock-like features. At the low plasma density, the initial pileup of He^2+^ at the channel wall is not sufficient to drive a shock, as shown in Fig. 6a, and hence unable to reflect protons, Fig. 6b.

Here, both helium and protons are accelerated by the same space-charge field, agreeing with the equal energy-per-charge experimental result shown in Fig. 2e. For protons, the shock reflection is mostly prominent only in the high-density regime (0.2 ${n}_{cr}$), similar to Fig. 2f. Background He^2+^ ions constitute a shock wall (Fig. 6c) from which a collective stream of protons is bounced (Fig. 6d). Consequently, these observations in 2D PIC simulations are in good agreement with the experimental finding, verifying that the transverse shock acceleration of protons is feasible in the high-density helium plasma driven by a PW 30 fs laser pulse. We also observe an oval-shape plasma channel in the case of Fig. 6b, which is qualitatively similar to the plasma channel observed in the experiments. It is also worth mentioning that for experiment-like plasma profile with lower densities (Fig. 6a), or flat plasma densities (Fig. 4), the channel profile observed in simulations was cylindrical.

## Discussion

The electrostatic collisionless shock acceleration observed in our experiment is the consequence of the NCD plasma driven by a femtosecond PW laser. The experimental result strongly indicates a clear transition of the acceleration mechanism from sheath acceleration to shock acceleration with the increase of plasma density. The distinct feature of sheath acceleration can be seen from the ratio of energy per charge in Fig. 2d, while the characteristics of shock acceleration are confirmed from the ratio of maximum velocity in Fig. 2e. The observation of shock acceleration at the higher density is because the high plasma density facilitates efficient and localized laser absorption and a stronger pileup of helium ions on the channel wall to strengthen the shock potential, leading to the shock formation by helium plasma and consequent shock acceleration of protons. The simulated proton phase space plot (Fig. 4e) shows clear bunching of the particles, representing modulation in the energy spectrum. However, the proton energy spectrum measured in the experiment does not have any feature of spectral modulation. At the moment, the exact mechanism of spectral smoothing for protons is not clearly known and will be investigated in a future study. In addition to the electrostatic shock acceleration of ions, the femtosecond PW laser-produced NCD plasma can be utilized to study the relativistic plasma dynamics relevant to high-energy–density physics.

While referring to the plasma density, the relativistic effects should be taken into the account. In our experiments, the normalized vector potential had a peak value of ${a}_{0}=7$ and the corresponding peak value of the Lorentz factor turns out to be $\gamma ={(1+{{a}_{0}}^{2}/2)}^{1/2}=5$. Therefore, the effective plasma density in the region of maximum laser intensity could drop by a factor of 5 (${n}_{e}^{eff}={n}_{e}/\gamma$). However, lowering of the plasma density due to the relativistic correction is only applicable inside the plasma channel, experiencing the highest laser intensity. As most of the dynamics related to the transverse ion acceleration is occurring at the boundary layer of the transversely expanding plasma channel, in these regions the relativistic factors can be ignored. Following these arguments, we can define our experiments to be in the “near-critical-density” NCD plasma regime.

Recently, some of the numerical works by Bulanov et al.^40^ and Nakamura et al.^41^ have shown the possibility of forward ion accelerations in the NCD plasma via magnetic vortex acceleration mechanism^42^. The ion acceleration occurs in the down ramp phase of the plasma density, where the expansion of magnetic vortex causes electrons to be expelled from the central region, which leads to the formation of the electric field responsible for ion acceleration^41^. Most importantly, for the magnetic vortex to be realized in the experiment, it was required that the plasma should have a narrow density down ramp of width only in tens of micrometer^41^. In contrast, the gas jet mediums used in our experiments has density down ramp with a scale of 100’s micrometer (see Fig. S1a). Due to the presence of an order of magnitude larger density down ramp, the magnetic vortex acceleration mechanism described by Bulanov^40^ and Nakamura^41^ could not be realized in our experiments. This is also consistent with the absence of any forward ion acceleration occurring in our experiment and simulation. Furthermore, our shadowgraphy diagnostics did not observe any vortex structure being formed in the plasma.

In the simulations, the longitudinal electrostatic field was too transient to sustain efficient forward ion acceleration and no forward ions were observed. This is in agreement with the experimental observation, where ion signal was recorded only in the transverse TPS and none in the forward TPS (Fig. 1). The ion density channel was also observed to expand mostly along the transverse direction, in agreement with ion measurement performed in the experiment. The expansion speed of the channel wall was found to be nearly 0.015c, for early few picoseconds, which is very close to the speed measured in the experiment. The corresponding energy of helium ions moving with the channel wall is around 0.5 MeV. This indicates that the helium ions with the maximum energy detected in the experiment originated from the plasma channel.

## Conclusion

In conclusion, we have investigated the transverse shock formation and ion acceleration in plasma driven by a femtosecond, PW laser. The multi-dimensional PIC simulations and the experimental data confirmed the mass-selective shock acceleration observed in experiments. We have also seen that by changing the plasma density, the plasma dynamics and shock formation could be altered. In the case of low-density plasma, the pileup of He^2+^ at the channel wall is not sufficient to drive an electrostatic shock and both helium and protons were accelerated for the similar electrostatic potential. However, in the case of a high plasma density, the localized efficient absorption of a laser pulse facilitated the high-energy–density conditions near the depletion region of the laser pulse, extending the charge-neutralization time scale of the plasma and allowed helium ions to gain radial momentum, and formed a transverse electrostatic shock, accelerating protons to twice higher velocity. The relativistic mass effect on the electron allows the electrostatic field in the plasma channel to be sustained on a long time scale facilitating transverse shocks to be developed even in the plasma driven by a femtosecond laser. Our study demonstrates that high-power femtosecond laser-produced NCD plasma could provide another pathway in the laboratory astrophysics to study the long-standing astrophysical collisionless shock problems.

## Methods

### Experimental setup

A PW laser pulse with a 30 J energy and 30 fs duration at the center wavelength of 800 nm, was focused by an F/10 spherical mirror onto a He gas jet to a 12 µm FWHM spot radius, corresponding to a typical intensity of $1\times {10}^{20}$ W cm^−2^ (normalized vector potential, ${a}_{0}=7$). The He gas jet was produced by a commercial high-density gas jet system (SL-GT-10) from Source Lab, having 400 µm cylindrical nozzle^33^. The laser pulse was focused at the center of the gas jet nozzle with the help of the top view diagnostics. Figure S1a shows the line out of the neutral gas density profile at two different height from the nozzle. For instance, at 1 mm away from the nozzle, the neutral gas density is about 10^20^ atoms/cm^3^. Therefore by changing the nozzle height with respect to the laser focus, the electron plasma density was scanned in the range of ${n}_{e}=2-4\times {10}^{20}$ cm^−3^. Here complete ionization of helium gas by the laser pulse is assumed as laser intensity ~ 1 × 10^20^ W cm^−2^, is four orders of magnitude stronger than required for the field ionization for ${He}^{2+}$ (8.8 × 10^15^ W cm^−2^). During the laser-plasma interaction, a strong 90° side scatter signal was also observed (Fig. 1b). The plasma scattering spectra were recorded by fiber-coupled optical spectrometers, installed along forward, backward and transverse directions of the laser propagation axis. The plasma density estimated by the Raman shift (${n}_{e}=2\times {10}^{20}{\mathrm{ cm}}^{-3}$) is in good agreement with the density estimated by the neutral gas measurements. The source of hydrogen could have originated either as an impurity in the gas delivery line or as a water vapor contamination. It was not possible to exactly quantify the fraction of hydrogen present in the gas, however, except helium lines, none of the hydrogen emission lines could be observed in the spectral range of 200–1100 nm. This indicates that the hydrogen fraction was negligible in comparison to the bulk helium gas. The spatial overlap of the laser and gas jet was verified using the side-view and top-view plasma imaging diagnostics, which allow the vertical and horizontal positioning of the beam, respectively. A 35 fs, 800 nm probe pulse, propagating orthogonally to the PW laser beam, is used to measure the plasma shadowgram at different time delays. The accelerated electrons were measured by a magnetic dispersion (0.4 T) based electron spectrometer (ESM), installed along forward and transverse direction of the PW laser beam. Two Thomson parabola spectrometers (TPS) for resolving the charge and kinetic energy of accelerated ions were installed along the forward and transverse direction of the PW laser axis. The parabolic ion traces, dispersed by the magnetic field (0.2 T) and electric field (2 kV/cm), were recorded using a micro-channel plate (MCP) with a phosphor screen imaged by a 16-bit CCD camera.

## Supplementary information

Supplementary Information.

## References

[1] Medvedev MV, Loeb A (1999). Generation of magnetic fields in the relativistic shock of gamma-ray burst sources. Astrophys. J..

[2] Medvedev MV, Fiore M, Fonseca RA, Silva LO, Mori WB (2005). Long-time evolution of magnetic fields in relativistic gamma-ray burst shocks. Astrophys. J..

[3] Silva LO (2003). Interpenetrating plasma shells: Near-equipartition magnetic field generation and nonthermal particle acceleration. Astrophys. J..

[4] Weibel ES (1959). Spontaneously growing transverse waves in a plasma due to an anisotropic velocity distribution. Phys. Rev. Lett..

[5] Forslund DW, Shonk CR (1970). Formation and structure of electrostatic collisionless shocks. Phys. Rev. Lett..

[6] Silva LO (2004). Proton shock acceleration in laser-plasma interactions. Phys. Rev. Lett..

[7] Fiuza F (2012). Laser-driven shock acceleration of monoenergetic ion beams. Phys. Rev. Lett..

[8] Fiuza F (2013). Ion acceleration from laser-driven electrostatic shocks. Phys. Plasmas.

[9] Haberberger D (2012). Collisionless shocks in laser-produced plasma generate monoenergetic high-energy proton beams. Nat. Phys..

[10] Pak A (2018). Collisionless shock acceleration of narrow energy spread ion beams from mixed species plasmas using 1 μm lasers. Phys. Rev. Accel. Beams.

[11] Tochitsky, S. *et al.* Laser-driven collisionless shock acceleration of ions from near-critical plasmas. *arXiv:2006.06892 [physics.plasm-ph]* (2020).

[12] Palmer CAJ (2011). Monoenergetic proton beams accelerated by a radiation pressure driven shock. Phys. Rev. Lett..

[13] Zhang H (2017). Collisionless shock acceleration of high-flux quasimonoenergetic proton beams driven by circularly polarized laser pulses. Phys. Rev. Lett..

[14] Willingale L (2006). Collimated multi-MeV ion beams from high-intensity laser interactions with underdense plasma. Phys. Rev. Lett..

[15] Chen SN (2017). Collimated protons accelerated from an overdense gas jet irradiated by a 1 μm wavelength high-intensity short-pulse laser. Sci. Rep..

[16] Lemos N (2012). Forward directed ion acceleration in a LWFA with ionization-induced injection. J. Plasma Phys..

[17] Huntington CM (2015). Observation of magnetic field generation via the Weibel instability in interpenetrating plasma flows. Nat. Phys..

[18] Fiuza F (2020). Electron acceleration in laboratory-produced turbulent collisionless shocks. Nat. Phys..

[19] Pukhov A, Sheng ZM, Meyer-ter-Vehn J (1999). Particle acceleration in relativistic laser channels. Phys. Plasmas.

[20] Mackinnon AJ (1999). Intense laser pulse propagation and channel formation through plasmas relevant for the fast ignitor scheme. Phys. Plasmas.

[21] Sarkisov GS (1997). Observation of the plasma channel dynamics and Coulomb explosion in the interaction of a high-intensity laser pulse with a He gas jet. JETP Lett..

[22] Krushelnick K (1999). Multi-MeV ion production from high-intensity laser interactions with underdense plasmas. Phys. Rev. Lett..

[23] Wei MS (2004). Ion acceleration by collisionless shocks in high-intensity-laser-underdense-plasma interaction. Phys. Rev. Lett..

[24] Sylla F (2012). Anticorrelation between ion acceleration and nonlinear coherent structures from laser-underdense plasma interaction. Phys. Rev. Lett..

[25] Lifschitz A (2014). Ion acceleration in underdense plasmas by ultra-short laser pulses. New J. Phys..

[26] Kahaly S (2016). Detailed experimental study of ion acceleration by interaction of an ultra-short intense laser with an underdense plasma. Sci. Rep..

[27] Macchi A (2007). Ion dynamics and coherent structure formation following laser pulse self-channeling. Plasma Phys. Control. Fusion.

[28] Danson CN (2019). Petawatt and exawatt class lasers worldwide. High Power Laser Sci. Eng..

[29] Sung JH, Lee SK, Yu TJ, Jeong TM, Lee J (2010). 0.1 Hz 1.0 PW Ti:sapphire laser. Opt. Lett..

[30] Fonseca RA (2008). One-to-one direct modeling of experiments and astrophysical scenarios: Pushing the envelope on kinetic plasma simulations. Plasma Phys. Control. Fusion.

[31] Fonseca, R. A. *et al.* OSIRIS: A three-dimensional, fully relativistic particle in cell code for modeling plasma based accelerators. In *Computational Science — ICCS 2002. ICCS 2002*. Lecture Notes in Computer Science (ed Sloot, P. M. A., Hoekstra, A. G., Tan, C. J. K., Dongarra, J. J.) vol 2331. 10.1007/3-540-47789-6_36 (Springer, Berlin, Heidelberg, 2002).

[32] Stockem A, Fiuza F, Bret A, Fonseca RA, Silva LO (2015). Exploring the nature of collisionless shocks under laboratory conditions. Sci. Rep..

[33] Sylla F, Veltcheva M, Kahaly S, Flacco A, Malka V (2012). Development and characterization of very dense submillimetric gas jets for laser-plasma interaction. Rev. Sci. Instrum..

[34] Pivovar LI, Tabuev VM, Novikov MT (1962). Electron loss and capture by 200–1500 kev helium ions in various gases. Sov. Phys. JETP.

[35] Sun G-Z, Ott E, Lee YC, Guzdar P (1987). Self-focusing of short intense pulses in plasmas. Phys. Fluids.

[36] Macchi A, Ceccherini F, Cornolti F, Kar S, Borghesi M (2009). Electric field dynamics and ion acceleration in the self-channeling of a superintense laser pulse. Plasma Phys. Control. Fusion.

[37] Kovalev VF, Bychenkov VY (2015). Dynamics of ponderomotive ion acceleration in a laser-plasma channel. Plasma Phys. Rep..

[38] Mori WB, Joshi C, Dawson JM (1988). Evolution of self-focusing of intense electromagnetic waves in plasmas. Phys. Rev. Lett..

[39] Sarri G (2010). Observation of postsoliton expansion following laser propagation through an underdense plasma. Phys. Rev. Lett..

[40] Bulanov SV, Dylov DV, Esirkepov TZ, Kamenets FF, Sokolov DV (2005). Ion acceleration in a dipole vortex in a laser plasma corona. Plasma Phys. Rep..

[41] Nakamura T, Bulanov SV, Esirkepov TZ, Kando M (2010). High-energy ions from near-critical density plasmas via magnetic vortex acceleration. Phys. Rev. Lett..

[42] Nakamura T, Mima K (2008). Magnetic-dipole vortex generation by propagation of ultraintense and ultrashort laser pulses in moderate-density plasmas. Phys. Rev. Lett..

