# Supplementary material for: Electrostatic shock acceleration of ions in near-critical-density plasma driven by a femtosecond petawatt laser
Source: Sci Rep. 2020 Oct 28;10:18452. doi: 10.1038/s41598-020-75455-1 (PMC7595239; doi:10.1038/s41598-020-75455-1)
Supplement: Supplementary file 1 — Supplementary Information. [file 41598_2020_75455_MOESM1_ESM.docx]

**Supplementary Information**

**Electrostatic shock acceleration of ions in near-critical-density plasma driven by a femtosecond petawatt laser**

Prashant Kumar Singh^1^, Vishwa Bandhu Pathak^1^, Jung Hun Shin^1^, Il Woo Choi^1,2^, Kazuhisa Nakajima^1^, Seong Ku Lee^1,2^, Jae Hee Sung^1,2^, Hwang Woon Lee^1^, Yong Joo Rhee^1^, Constantin Aniculaesei,^1^ Chul Min Kim^1,2^, Ki Hong Pae^1,2^, Myung Hoon Cho^1^, Calin Hojbota^1,3^, Seong Geun Lee^1,3^, Florian Mollica^4, 5^, Victor Malka^5.6^, Chang-Mo Ryu^1^, Hyung Taek Kim^1,2*^, and Chang Hee Nam^1,3**^

[*htkim@gist.ac.kr](mailto:*htkim@gist.ac.kr) and [**chnam@gist.ac.kr](mailto:**chnam@gist.ac.kr)

^1^Center for Relativistic Laser Science, Institute for Basic Science (IBS), Gwangju 61005, Republic of Korea

^2^Advanced Photonics Research Institute, Gwangju Institute of Science & Technology (GIST), Gwangju 61005, Republic of Korea

^3^Department of Physics and Photon Science, GIST, Gwangju 61005, Republic of Korea

^4^Amplitude Laser Group, 91090 Lisses, France

^5^Laboratoire d’Optique Appliquée, ENSTA-ParisTech, Ecole Polytechnique, 828 Boulevard des Marechaux, 91762 Palaiseau CEDEX, France.

^6^Weizmann Institute of Science, P.O. Box 26, Rehovot 76100, Israel.

**A. Plasma density estimation and electron spectrum**

The plasma density in the interaction volume was estimated from the neutral density measurement and from the Raman scattering signal measured with a visible spectrometer. The neutral gas density profile is shown for two heights from the nozzle in Fig. S1(a). At 1000 µm away from the nozzle, the peak neutral gas density is about $1\times{10}^{20}$ cm^-3^. By changing the laser focusing position from the nozzle, the electron density was varied in the range of$n_{e}=(2-4)\times{10}^{20}{cm}^{-3}$. Here the complete ionization of helium by the laser pulse with an intensity of 1×10^20^ W cm^-2^ occurred, since the laser intensity was about four orders of magnitude stronger than that required for the optical field ionization of He^+^ (8.8×10^15^ W cm^-2^). During the laser-plasma interaction, strong Raman scattering signal was also observed (Fig. S1(b)). The plasma scattering spectra were recorded by fiber-coupled optical spectrometers, installed along forward, backward and transverse directions of the laser propagation axis. The plasma density estimated by the Raman shift ($n_{e}=2\times{10}^{20}{cm}^{-3}$) was in good agreement with the density estimated by the neutral gas density measurement. Since only helium emission lines, without hydrogen lines, could be observed in the spectral range of 200 nm – 1100 nm, the hydrogen fraction was minimal in comparison to the bulk helium gas and the plasma dynamics was determined mainly by the helium plasma.


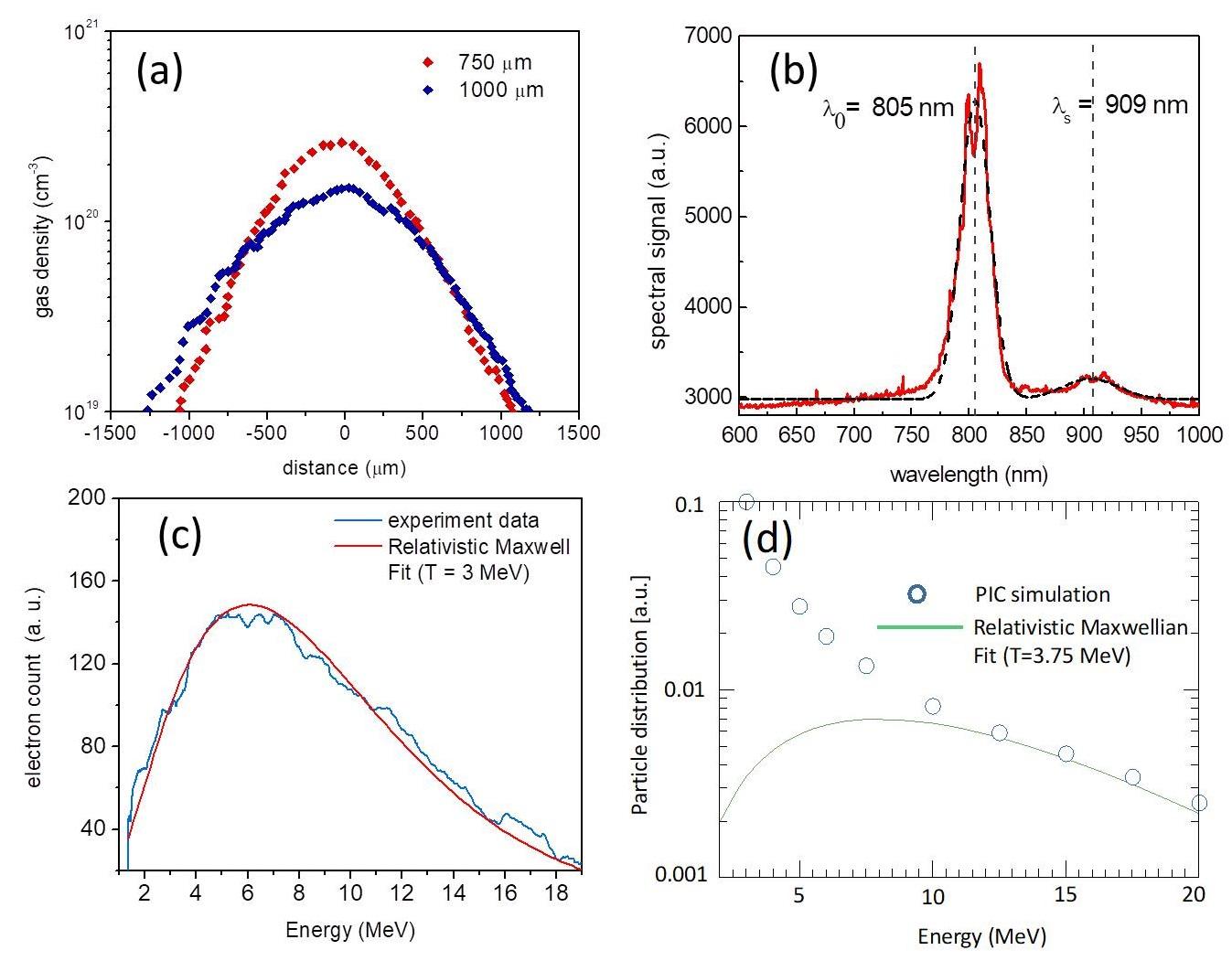


**Figure S1.** (a) Line out of the neutral gas density profile at a height of 750 µm and 1000 µm away from the nozzle tip. (b) Raman scattering signal observed from the transverse direction, corroborating with the plasma density. Two dotted lines in (b) represent the central peak position for the fundamental (805 nm) and the scattered light (909 nm). The kinetic energy spectrum of forward electrons measured at plasma density of$n_{e}= 4.2\times{10}^{20}{cm}^{-3}$ in experiment (c) and in PIC simulation (d).

Relativistic electrons with kinetic energy up to 20 MeV were detected along the forward direction at an angle 10º by an electron spectrometer with the magnetic field 0.4 T, as shown in Fig. S1(c). The electron spectrum was fitted to relativistic Maxwellian distributions with an electron temperature of 3 MeV. The electron spectrum was fitted to the relativistic Maxwellian distribution with an electron temperature of 3 MeV. In addition, the 2D PIC simulation, having electron temperature of 3.75 MeV (Fig. S1(d)), is also in a good agreement with the experimental observation (Fig. S1(c)).*”*

**B. Charge exchange process**

The helium ion spectra, shown in Fig. 2, indicate that the strong charge exchange process occurred during the plasma evolution after the generation of fully ionized helium plasma. The spectral image of He^+^ and He^2+^ ions recorded with the transverse TPS shows that the He^+^ trace is much stronger than the He^2+^ trace. A constant velocity line drawn from the zero deflection point is shown to connect the high energy ends of both ion traces, indicating that the ion energy is the same for both charge states. A quantitative comparison is plotted in Fig. S2(b), showing the kinetic energy spectra of He^+^ and He^2+^, for a typical laser shot. In the low energy range (< 300 keV), the He^+^ flux is larger than the He^2+^ by more than one order of magnitude, whereas in the high energy part this difference gets smaller with energy. However, in agreement with the constant velocity line, the maximum energy of both He^+^ and He^2+^, is similar (0.9 MeV). The laser intensity employed, 1×10^20^ W cm^-2^, being nearly four orders of magnitude higher than required for the field ionization of He^+^ (8.8×10^15^ W cm^-2^), ensures the complete ionization of helium. He^+^ can only be present very far from the main interaction region, from which it cannot be accelerated to such high energies observed in the experiments. These arguments indicate the strong possibility that the energetic He^+^ ions are produced via the strong charge-exchange/recombination of accelerated He^2+^ ions with the surrounding neutral gas. We examined the charge exchange process from the rate equations of helium ions for dominant electron-capture and loss processes:

$\frac{d{He}^{2+}}{dZ}$ $=N_{0}(-\sigma_{21}{He}^{2+}+\sigma_{12}{He}^{+})$ (i)

$\frac{d{He}^{+}}{dZ}$ $=N_{0}(-\sigma_{10}{He}^{+}+\sigma_{21}{He}^{2+}+\sigma_{01}{He}^{0}-\sigma_{12}{He}^{+})$ (ii)

$\frac{d{He}^{0}}{dZ}$ $=N_{0}(-\sigma_{01}{He}^{0}+\sigma_{10}{He}^{+})$ (iii)

where ${He}^{0}$, ${He}^{+}$ and ${He}^{2+}$are the number densities of neutral, singly charged and doubly charged helium, $N_{0}$ is the average gas density, $\sigma_{01}$ (${He}^{0}\to$ ${He}^{+}$), $\sigma_{10}$ (${He}^{+}\to$ ${He}^{0}$), $\sigma_{12}$ (${He}^{+}\to$ ${He}^{2+}$), $\sigma_{21}$ (${He}^{2+}\to$ ${He}^{+}$) are the cross-sections for the electron capture or the electron loss process. For an average gas density $N_{0}={10}^{20} {cm}^{-3},$ thickness$L=0.1 cm$, and typical cross-section $\sigma={10}^{-16} {cm}^{2},$the factor ${(N}_{0}\times L\times\sigma\gg1)$ indicates that the partial densities of fast ions are in equilibrium and the left-hand sides of equation (i), (ii) and (iii) can be close to zero. Therefore, the relative number density between helium species is related to the relative cross-section, which gives ${He}^{+}=$ ${{(\sigma}_{21}/\sigma_{12})\times He}^{2+}$ and ${He}^{0}=$ ${{(\sigma}_{10}/\sigma_{01})\times He}^{+}$.


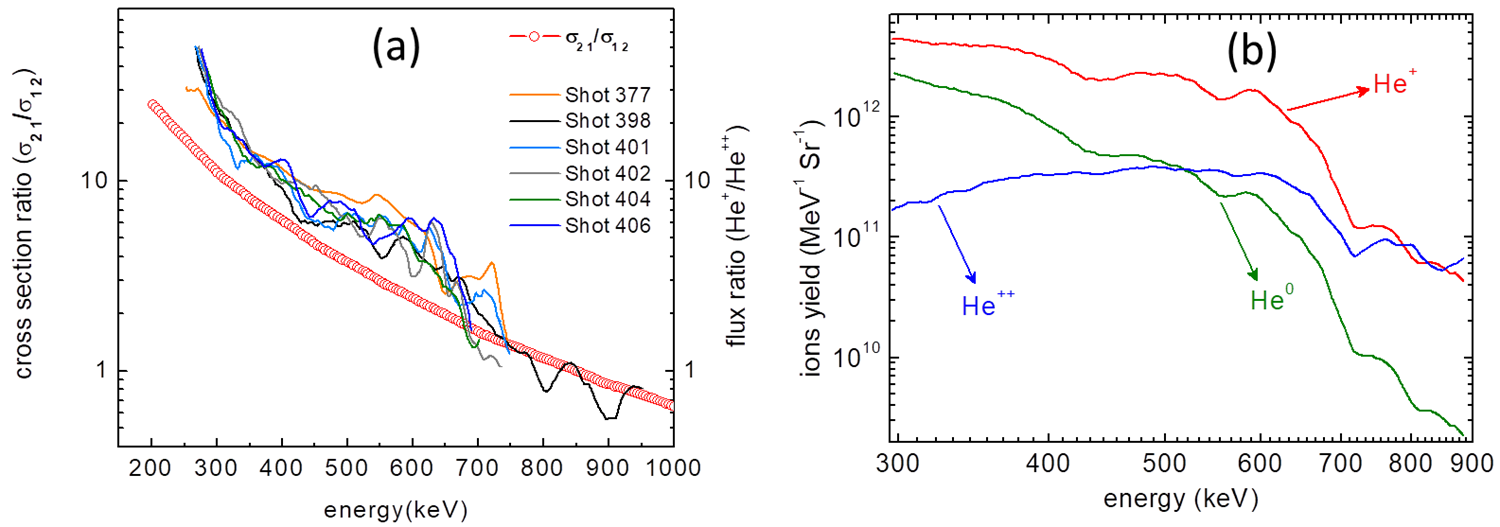


**Figure S2.** (a) Flux ratio of He^+^ ions to He^2+^ ions for different laser shots, given as a function of ion energy. The flux ratio is compared with the ratio of the electron capture to the electron loss cross-sections. (b) Measured kinetic energy spectra of He^2+^ and He^+^ ions, along with the neutral helium spectrum estimated from the charge exchange cross-section ratio.

The ratio of the electron capture to the electron loss ($\sigma_{21}/\sigma_{12}$), taken from Pivovar^34^ et al., is compared with the flux ratio of He^+^ and He^2+^, obtained from different laser shots, as shown in Fig. S2(a). They show good agreement for the entire energy range of measurement (200- 800 keV). It is thus a good indication that He^+^ ions are produced via the strong charge-exchange process between accelerated He^2+^ ions with the surrounding neutral gas. Furthermore, this analysis can be extended to estimate the density of neutral helium from the interaction, although in the experiment it was not measured. The neutral helium density, given as ${He}^{0}=$ ${{(\sigma}_{10}/\sigma_{01})\times He}^{+}$, is plotted in Fig. S2(b) along with ${He}^{+}$ and ${He}^{2+}$. ${He}^{0}$dominates over ${He}^{2+}$ till nearly 500 keV, after which it falls down due to very small cross-section. The above estimate suggests that the near-critical-density plasma provides an efficient way of producing neutral or lower charge ions through the charge exchange process.

**C. Density-dependent plasma dynamics**

Figures S3(a-c) and S3(d-f) show the temporal evolution of the plasma, corresponding to two different plasma density of $2.3\times{10}^{20}{cm}^{-3}$and $4.2\times{10}^{20}{cm}^{-3}$, respectively. For the lower plasma density ($2.3\times{10}^{20}{cm}^{-3}$), the laser absorption was not localized and multiple plasma filaments were observed (Fig. S3(a)). On the other hand, for two times higher plasma density ($4.2\times{10}^{20}{cm}^{-3}$), the laser energy depletion occurred in a very small volume, indicated by much smaller and bright plasma scattering image in Fig. S3(d). The localized absorption of laser energy created a high-energy-density region, along with rapid evacuation of electrons, and later on, followed by ions.


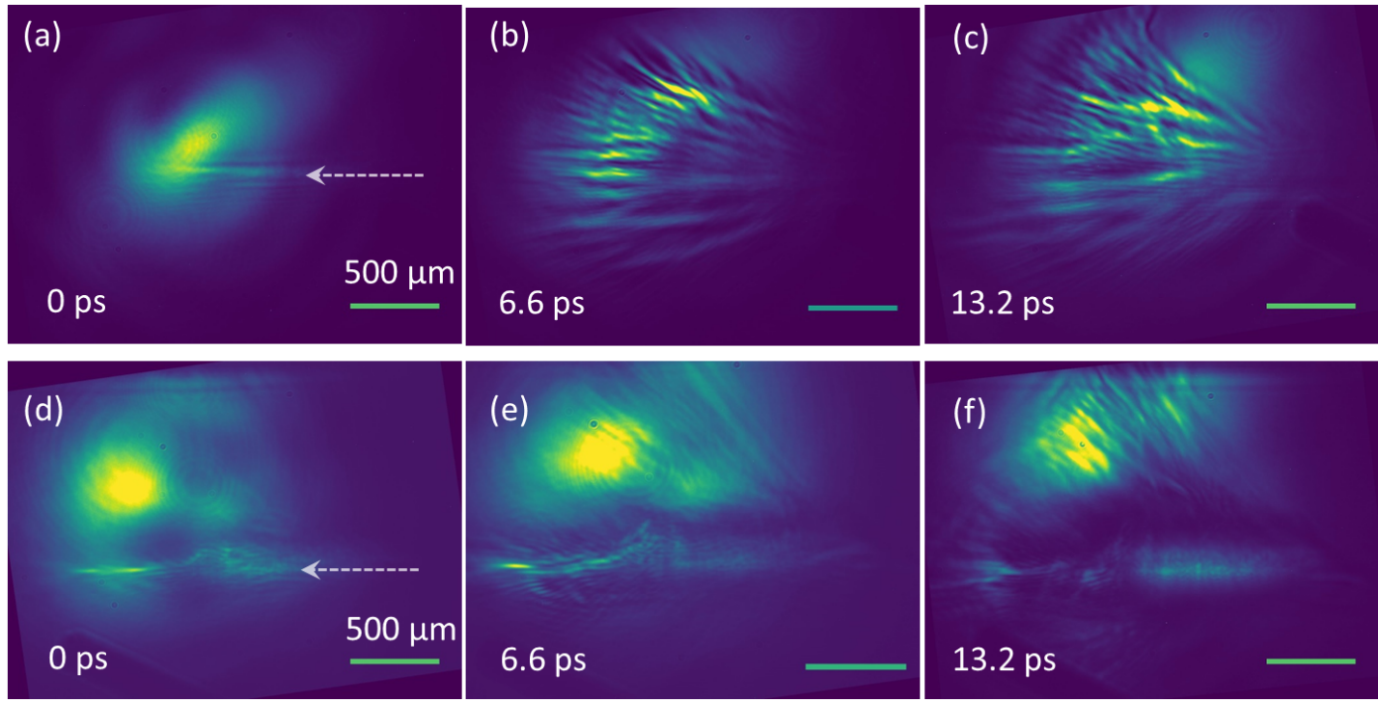


**Figure S3. Density-dependent plasma dynamics.** (a), (b)and (c) Shadowgram of plasma at the temporal delay of 0.0 ps, 6.6 ps and 13.2 ps, respectively, for the plasma density of *n_e_* = 2.3×10^20^ cm^-3^. (d), (e) and (f) Shadowgrams of plasma at the temporal delay of 0.0 ps, 6.6 ps and 13.2 ps, respectively, for the plasma density of *n_e_* = 4.5×10^20^ cm^-3^. The white arrows in (a) and (d) show the propagation direction of the laser.
